# Systematic review and meta-analysis comparing educational and reminder digital interventions for promoting HPV vaccination uptake

**DOI:** 10.1038/s41746-023-00912-w

**Published:** 2023-08-29

**Authors:** Nutthaporn Chandeying, Therdpong Thongseiratch

**Affiliations:** 1 Gynecologic Oncology Unit, Department of Obstetrics and Gynecology, Faculty of Medicine, Vajira Hospital, Navamindradhiraj University, Bangkok, Thailand; 2 Child Development Unit, Department of Pediatrics, Faculty of Medicine, Prince of Songkla University, Songkhla, Thailand

**Keywords:** Health care, Infectious diseases, Paediatric research

## Abstract

Global Human papillomavirus (HPV) vaccination rates remain low despite available WHO-approved vaccines. Digital interventions for promoting vaccination uptake offer a scalable and accessible solution to this issue. Here we report a systematic review and meta-analysis examining the efficacy of digital interventions, comparing educational and reminder approaches, for promoting HPV vaccination uptake (HVU). This study also identifies factors influencing the effectiveness of these interventions. We searched PubMed, PsycInfo, Web of Science, and the Cochrane Library from each database’s inception to January 2023. Three raters independently evaluate the studies using a systematic and blinded method for resolving disagreements. From 1929 references, 34 unique studies (281,280 unique participants) have sufficient data. Client reminder (OR, 1.41; 95% CI, 1.23–1.63; *P* < 0.001), provider reminder (OR, 1.39; 95% CI, 1.11–1.75; *P* = 0.005), provider education (OR, 1.18; 95% CI, 1.05–1.34; *P* = 0.007), and client education plus reminder interventions (OR, 1.29; 95% CI, 1.04–1.59; *P* = 0.007) increase HVU, whereas client education interventions do not (OR, 1.08; 95% CI, 0.92–1.28; *P* = 0.35). Digital intervention effectiveness varies based on participants’ gender and the digital platform used. Interventions targeting male or mixed-gender participants demonstrate greater benefit, and reminder platforms (SMS, preference reminders, or electronic health record alerts) are more effective in increasing HVU. Digital interventions, particularly client and provider reminders, along with provider education, prove significantly more effective than client education alone. Incorporating digital interventions into healthcare systems can effectively promote HPV vaccination uptake. Reminder interventions should be prioritized for promoting HVU.

## Introduction

Cervical cancer is the fourth most common cancer among women worldwide^1^, with an estimated 600,000 new cases diagnosed each year^2^. The majority of cases occur in developing countries, where access to preventive measures is often limited^3^. Preventive measures for cervical cancer include vaccination against human papilloma virus (HPV), regular screening tests, and safe sexual practices^4^. Scale-up of HPV vaccination uptake (HVU) for adolescent girls will have a significant effect in preventing cervical cancer and its associated mortality^5^. The World Health Organization has set the goal of having 90% of girls vaccinated against HPV by the time they turn 15 as part of its global strategy to eliminate cervical cancer. More than 45 million deaths from cervical cancer and other HPV-related cancers, such as vaginal cancer, laryngeal cancer, and anal cancer, could be prevented if the WHO’s 2030 target for delivering prophylactic HPV vaccination were met^6^.

Yet, the coverage of HPV vaccination varies widely around the world with 33.6% in developed regions, but only 2.7% in less developed regions^7,8^. There are several factors that can influence the HVU. These include the availability and accessibility of the vaccine, the cost of the vaccine, the recommendations and policies of national and local health authorities, and cultural and societal attitudes towards vaccination^9–11^.

School-based vaccination programs^12,13^, education about HPV and the vaccine at the outpatient clinic^14,15^, reminder letters to students and parents about upcoming vaccination appointments^16,17^, and incentives for vaccination can promote HVU^18,19^. However, these existing strategies face several obstacles, including undervaluation, misunderstanding, attitudinal barriers^20–23^, structural barriers such as scheduling challenges^12,13^, and expensive incentives^16,17,20^. Digital interventions leveraging technology and online platforms offer solutions to these challenges^24–27^.

Digital interventions can be utilized to enhance vaccination uptake through both client and provider-based interventions. For clients, these interventions can furnish education about the importance of vaccination^26^ and dispel prevalent myths^27^. They can also send reminders and alerts for upcoming vaccination appointments^17^, track vaccination records, and facilitate the easy scheduling of vaccination appointments^28,29^. For healthcare providers, digital tools not only support in delivering vaccination services but also provide education through websites or webinars^30^. This education can include clinical decision support tools that aid in making informed decisions about vaccination^31^, and methods for remotely monitoring patients’ vaccination status^32,33^.

Previous systematic reviews have identified several commonly used interventions for increasing vaccination rates, including client education, client reminders, client education plus reminders, provider education, and provider reminders. These interventions have been widely implemented across various health systems and incorporate digital tools such as mobile phone messaging, applications, websites, and social media platforms. Their effectiveness in improving vaccination uptake has been well-documented^14–17,22,25,26^. These methods have been rigorously tested through Randomized Clinical Trials (RCTs)^34–43^, and although the results are promising, the implementation of guidelines for health promotion practice presents challenges. Specifically, these challenges arise from the lack of a direct meta-analytic comparison of these five key interventions, complicating the clear assessment of their individual effectiveness.

Here we present a systematic review and meta-analysis with the primary objectives to: (1) obtain a comprehensive estimate of effect sizes for digital client education (i.e., website, social media, or texting), client reminder (i.e., SMS, email, or both), the combination of client education and reminder, provider education (i.e., webinar or email), and provider reminder (i.e., electronic medical records or texting); (2) determine which of these five digital interventions significantly increase HVU; and (3) compare the magnitudes of increasing HVU produced by each digital intervention type. The secondary objective is to identify independent variables associated with digital intervention efficacy for promoting HVU. This data can enhance a customized digital intervention strategy and inform future research.

## Results

### Study selection and characteristics

#### Studies

**Fig. 1:**
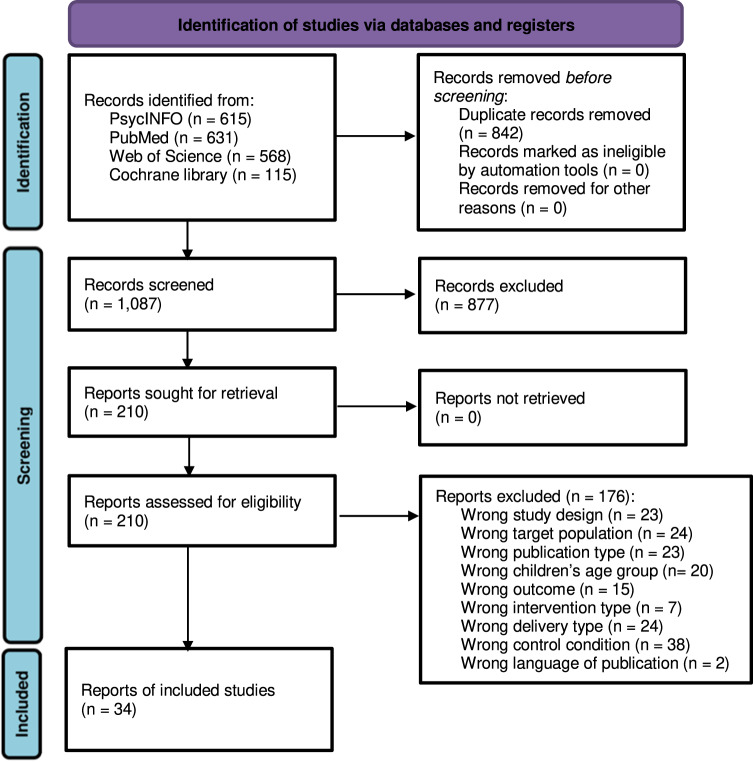
PRISMA flow diagram showing the study selection process.

We selected 1087 titles and abstracts for initial review. We selected 210 articles for full review. One hundred and seventy-six articles did not meet inclusion criteria (e.g., nonrandomization, ineligible intervention method) and were eliminated. Ultimately, we analyzed 34 unique studies (Supplementary Table 1) and calculated 41 effect sizes (6 articles had multiple intervention arms). Of these 41 effect sizes, 11 evaluated client education interventions, 9 evaluated client reminder interventions, 9 evaluated the combination of client education and reminder interventions, 4 evaluated provider education interventions, and 8 evaluated provider reminder interventions. Figure 1 displays the PRISMA study selection flowchart.

#### Participants

A total of 281,280 unique participants were drawn from the 34 included studies^27,32,34–65^. Twenty-five studies were conducted on parents and/or adolescents (73.5%), while the remaining studies were conducted on healthcare providers. Thirteen studies only included women, while three only included men. In all studies, the mean age of participants was 15 (range: 9–45). All included studies were conducted in high-income countries (*k* = 28 for the United States, *k* = 1 for Australia, *k* = 1 for Israel, *k* = 1 for the Netherlands, and *k* = 1 for Japan) and an upper-middle income country (*k* = 2 for China).

#### Intervention and control conditions

The average duration of interventions was 8 months (range, 1–24). Concerning control conditions, 23 studies (66.7%) utilized usual care or no intervention, while 11 studies (32.3%) utilized an attention or education control. Five studies on client education examined client education via website, three DVDs or Videos, one Facebook, and one email or SMS. Seven studies on client reminders evaluated SMS, email, autodialed phone, or Facebook messenger, and two evaluated an electronic health record alert system. Four combined intervention studies (client education plus client reminder) tested SMS or email, two websites, and two mobile applications. Two studies on provider education examined provider education via webinar and website. An electronic health record reminder system was evaluated in five provider reminder studies. Twenty-eight studies employed a traditional 2-arm RCT design (i.e., intervention vs. control), five studies employed a 3-arm RCT design (i.e., intervention 1 vs. intervention 2 vs. control), and one study employed a 4-arm RCT design (i.e., intervention 1 vs intervention 2 vs intervention 3 vs control). The primary outcomes of the 30 included studies were vaccine initiation, whereas the primary outcomes of the remaining studies (*k* = 4) were vaccine series completion. The majority (*k* = 27) of primary study outcomes utilized provider-validated measures or electronic medical records that were evaluated at baseline and post-assessment. Supplementary Table 1 provides a comprehensive summary of all studies included.

#### Quality of studies

Supplementary Figs. 1, 2 presents the methodological quality of the 34 included studies. Overall, no study satisfied all ROB 2.0 criteria and was deemed low risk for all five domains. More than half of the studies were judged as having some concerns as there were issues with the randomization process and deviation from the intended intervention. Twelve trials failed to provide information on the generation and concealment of random allocation sequences. Nine studies lacked information regarding blinding of participants and interventionists. Due to the nature of certain interventions, it was not possible to blind interventionists. Such occurrences in clinical trials were viewed as contextual deviations that were unlikely to affect trial outcomes. The ROB 2.0 algorithm determined that these trials posed a low risk of bias.

## Main results

### Vaccination uptake by intervention type

**Fig. 2:**
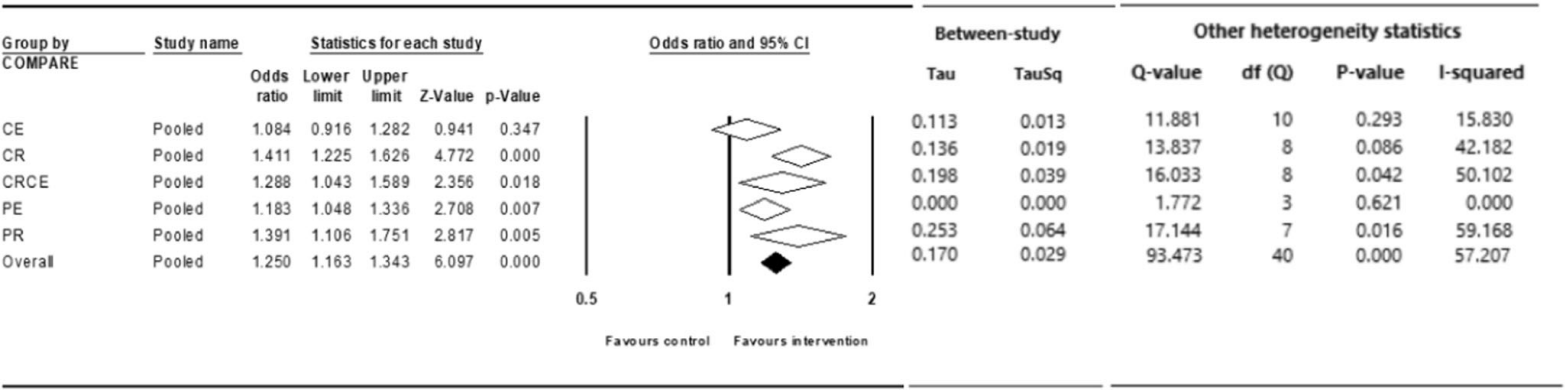
Meta-analysis results of the effect of digital interventions on Human papillomavirus vaccination uptake across all interventions, client education (CE), client reminder (CR), client education plus reminder (CRCE), provider education (PE), provider reminder (PR) interventions.

We found significant increase in HVU (odds ratio [OR], 1.25; 95% CI, 1.16–1.34; *P* < 0.001; I^2^ = 57%) across all 41 comparisons from 34 studies, including all 5 intervention types (i.e., client education [*k* = 11], client reminder [*k* = 9], client education plus reminder [*k* = 9], provider education [*k* = 4], and provider reminder [*k* = 8]). Studies that intervened with client reminder demonstrated the largest overall improvements in HVU, with significant effects (OR, 1.41; 95% CI, 1.23–1.63; *P* < 0.001; I^2^ = 42%). Studies using provider reminder interventions exhibited similar improvements in HVU (OR, 1.39; 95% CI, 1.11–1.75; *P* = 0.005; I^2^ = 59%). Studies that delivered the combination of client education plus reminder interventions also exhibited similar improvements in HVU (OR, 1.29; 95% CI, 1.04–1.59; *P* = 0.007; I^2^ = 50%). Provider education interventions yielded significant but small improvements in HVU (OR, 1.18; 95% CI, 1.05–1.34; *P* = 0.007; I^2^ = 0%). Client education interventions yielded non-significant improvements in HUV (OR, 1.08; 95% CI, 0.92–1.28; *P* = 0.35; I^2^ = 15%). Comparisons across all 5 intervention types revealed that client reminder, client education plus reminder, provider education, and provider reminder interventions produced significantly greater improvements in HVU compared with client education interventions. (Fig. 2 and Supplementary Fig. 3 depict forest plots).

### Independent variables associated with intervention effectiveness

We tested whether each of 8 variables listed in the Methods section was associated with the effectiveness of all 5 intervention types for improving HVU per their effect sizes (for all data and P values, see Table 1). Results suggest that intervention effectiveness is associated with 2 variables: gender (only female, only male, or mixed), and digital platform used. Although improvements in HVU were reported by both genders, studies targeting only male or mixed gender participants reported the greater benefit. Reminder platforms (SMS, preference reminders, or electronic health record alert) had a greater impact on increasing HVU compared to education platforms (website, webinar, or Facebook) alone.

**Table 1:** Factors associated with digital intervention effectiveness in promoting Human papillomavirus vaccination uptake.

Variables	Odd ratio (95%CI)	*P*-Value	No. of Effect Sizes
Age group	Q (df = 2) = 0.227	0.89	
Children and adolescent (9–18 years of age)	1.13 (1.08–1.18)	< 0.0001	27
Young adults (18–26 years of age)	1.23 (1.04–1.45)	0.02	12
Children, adolescents and young adults (9–18 years of age)	1.03 (0.37–2.84)	0.95	2
Gender	Q (df = 2) = 10.51	0.005	
Only female	1.06 (1.01–1.12)	0.02	16
Only male	2.30 (1.52–3.48)	< 0.001	4
Mixed	1.30 (1.21–1.41)	< 0.001	21
Intervention target	Q (df = 4) = 5.51	0.24	
Only patients	1.35 (1.10–1.65)	0.004	11
Only parents	1.10 (1.04–1.15)	< 0.001	13
Only providers	1.21 (1.10–1.32)	< 0.001	12
Parents and patients	1.46 (0.75–2.84)	0.27	4
Parents and providers	1.90 (1.33–2.70)	< 0.001	1
Platform	Q (df = 4) = 30.34	< 0.001	
Primary reminder delivery mode, reminder intervention			
SMS	1.39 (1.23–1.55)	< 0.001	6
Preference reminders (SMS or email or Facebook messenger or autodial phone)	1.33 (1.07–1.65)	0.01	7
Electronic health record	1.39 (1.24–1.56)	< 0.001	11
Primary education delivery mode, education intervention			
Website	1.07 (0.99–1.15)	0.10	9
Video	1.55 (0.92–2.60)	0.10	3
Application	1.53 (0.97–2.42)	0.07	2
Webinar	1.27 (0.96–2.69)	0.10	2
Facebook	1.01 (0.95–1.08)	0.73	1
Intervention site	Q (df = 1) = 0.039	0.844	
Clinic	1.25 (1.15–1.36)	< 0.001	17
Non-clinic	1.11 (1.05–1.16)	< 0.001	24
Vaccination uptake outcome	Q (df = 1) = 1.37	0.24	
Uptake	1.12 (1.08–1.17)	< 0.001	35
Series completion	1.51 (1.22–1.88)	< 0.001	4
Minority participant	Q (df = 1) = 1.37	0.87	
General participant	1.12 (1.07–1.17)	< 0.001	31
Minority participant	1.38 (1.18–1.62)	< 0.001	8
Control condition	Q (df = 1) = 0.32	0.57	
Usual control	1.12 (1.07–1.17)	< 0.001	29
Specific component (i.e., attention, education)	1.29 (1.13–1.47)	< 0.001	12

The following variables were not associated with intervention effectiveness: age group, intervention targets (patients, parents, or providers), intervention site (clinic or non-clinic), vaccination uptake outcome (initiation and completion), minority participants, or type of control conditions. Patients of all ages equally experienced improvements in HVU. Interventions targeting patients, parents or providers were equally effective for increasing HVU. However, the combination of parents plus provider intervention target was most effective. Participants were also equally likely to report improvements in HVU regardless of intervention site, vaccination uptake outcome, minority participants, and type of control conditions.

### Small study effects and publication bias

**Fig. 3:**
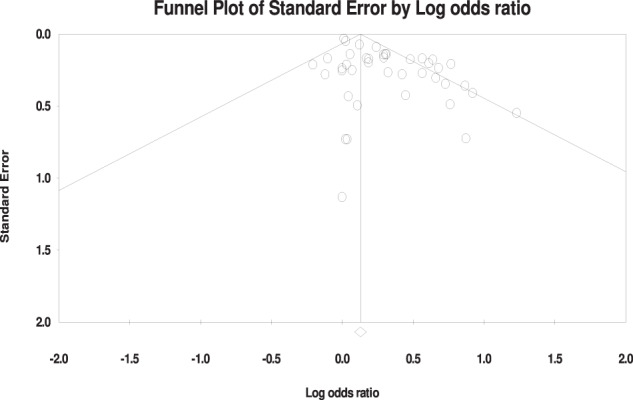
Funnel plot of effect sizes around the mean effect size for human papillomavirus vaccination uptake.

Visual inspection of the funnel plot (Fig. 3) showed no evidence for publication bias. Distribution of effect sizes was fairly symmetrical. Most effect sizes fell in the funnel; effect sizes falling outside the funnel did so symmetrically. Egger’s regression intercept test was statistically significant (intercept = 1.35, 95% CI 0.78–1.92, *p* < 0.001). It suggests that publication bias may be influencing the observed results. The Duval and Tweedie’s trim and fill procedure was conducted to assess publication bias. Fourteen missing studies were imputed during the analysis to account for potential bias. The pooled odds ratio for the included studies was 1.14 (95% CI 1.09–1.18), indicating a significant association. After adjustment with imputed studies, the odds ratio was slightly attenuated to 1.08 (95% CI: 1.03–1.12). No evidence of a small study effect was observed, and the effect of the interventions remained statistically significant after adjusting for potential publication bias. In conclusion, although the visual inspection of the funnel plot showed no evidence of publication bias and a symmetrical distribution of effect sizes, the statistically significant result of Egger’s regression intercept test suggests the influence of publication bias. However, after conducting the Duval and Tweedie’s trim and fill procedure, the adjusted analysis yielded a slightly attenuated odds ratio, while maintaining statistical significance. Although publication bias may have affected the observed results, no evidence of a small study effect was found.

## Discussion

This meta-analysis is, to our knowledge, the most comprehensive and rigorous examination to date of the efficacy of digital interventions in promoting HVU. This meta-analysis is also the first to calculate odds ratios across 34 RCTs investigating the efficacy of the five most common intervention categories for promoting vaccination uptake. Our findings indicate that digital interventions are effective in promoting HVU. Specifically, client reminder, client education plus reminder, provider education, and provider reminder digital interventions are beneficial in promoting HVU, whereas client education strategies studied to date are ineffective.

The magnitude of our effects, particularly for reminder interventions (OR = 1.41 for client reminder and OR = 1.39 for provider reminder), is not substantially different from the effect sizes identified in meta-analyses of face-to-face clinic-based (OR = 1.14) or school-based (OR = 1.46) interventions for promoting HVU^66^. These similarities suggest that digital and face-to-face interventions might be similar in their effectiveness.

Client education without reminder system did not improve HVU, which is consistent with findings from the literature on face-to-face interventions. For example, Ampofo and colleagues reported no association between school-based education interventions and HVU in a small meta-analysis of interventions^67^. Similarly, Mohamed and colleagues investigated the idea that parent reminder interventions might be more effective in increasing HVU than non-reminder parental interventions in a subgroup analysis^68^. They examined 13 RCTs and found a statistically significant increase in HVU of 19% for studies that included reminders (e.g., postal, telephone, and digital reminders) but no statistically significant difference for education interventions that did not include reminders. We hypothesized that improving HPV vaccination knowledge and attitude is not directly linked to HPV vaccination behavior. There are likely to be differences in the composition of HPV vaccination behavior in direct and indirect studies that might affect these findings. This finding is also comparable to that of a previous meta-analysis focusing primarily on early childhood vaccination uptake that found that reminder systems were efficacious for improving vaccination uptake^17^. The present meta-analysis however, conducted separate meta-analyses for digital interventions specific to HPV vaccination and found that reminder interventions were more effective than education interventions. Hence digital reminders are recommended for use in primary care to improve HVU.

Moreover, we found that client reminder and client education did not have an additive impact on HVU; their combined use was not associated with a greater increase HVU than the use of client reminder alone. These combinations could be counterproductive owing to insufficient reminder prescriptions and added complexity and time demands leading to reduced adherence. The effect of education intervention for increasing vaccination uptake was driven by two factors: (a) the study occurring in a low- and middle-income country (LMIC) and (b) parents having a discussion with a professional expert, rather than receiving self-directed information^67^. The characteristics of the participants (conducted in high income countries) and education interventions (almost all are self-directed) reported in the included studies may explain the non-significant effect of digital education interventions. Interventions that raise the basic level of parental knowledge are therefore more effective in areas where understanding and awareness is low compared to countries where it is comparatively higher and educational barriers to HPV vaccination may be more subtle and linked to vaccine belief^23,26,67^. The utility of educational strategies within standard practice may be further questioned when examined alongside the results of trials that provided parents with both vaccination education and reminders. This finding has implications for policy as it suggests that reminder systems may be sufficient facilitators of HPV vaccination uptake.

The identification of family-level factors associated with digital intervention effectiveness on promoting HVU can help to streamline the vaccination promotion strategies by prioritizing for or matching interventions to those who will benefit most from digital interventions. Similarly, identifying program-level factors helps to highlight the barriers and components of the interventions that are important for effectiveness, and thus can inform and improve the future development and delivery of digital interventions. This meta-analysis is the first to demonstrate that the effectiveness of digital vaccination promotion interventions is related to one family-level factor (gender) and one program-level factor (digital platform). Studies targeting only male participants reported the greatest benefit. It may be that digital education and reminder interventions are as relevant for the unique challenges faced by male adolescents and parents of male adolescents (lower knowledge and awareness)^69^. Interventions were more effective for increasing HVU when delivered using reminder platforms (SMS, preference reminders, or electronic health record alert). It has been suggested that educational platforms are not as important for digital intervention effectiveness as it is for face-to-face vaccination promotion intervention, and that reminder platform may be sufficient for digital interventions. However, it is possible that certain program-level factors only moderate digital intervention effects for certain family-level factors, thus necessitating a further analysis of their interplay.

The non-significance of target (patients, parents or providers) as a moderator indicates that even digital interventions specifically designed for adolescent’s needs and/or improve parent or provider awareness have a benefit of promoting HVU. Future research is warranted to examine the potential pathways, including whether increasing awareness of providers may lead to increase vaccination uptake and/or whether increases in patient’s awareness may increase provider vaccine prescription. Digital interventions did not yield significant effects if the interventions targeted on children through young adults (9–26 years), suggesting that more specific intervention target, such as children or adolescents, should be developed.

Although our study primarily focused on the efficacy of digital interventions, it is important to consider the cost-effectiveness of implementing these strategies. Some studies have suggested that digital interventions, particularly those involving client reminders and provider reminders, can be more cost-effective than traditional, face-to-face interventions^70^. Digital reminders can reduce costs associated with printing and mailing, and can be more easily scaled up for large populations. Furthermore, provider reminders that leverage electronic health record systems can be integrated into existing workflows with minimal additional investment^71^. However, we recommend future research to conduct a comprehensive cost-effectiveness analysis of these digital interventions for promoting HVU, to better inform policy decisions and healthcare system integration.

Our study highlights some limitations that must be considered. There was considerable variation in the control groups used across the studies, with some employing usual controls and others using specific attention control conditions. This variation has led to greater differences between the studies, complicating direct comparisons. Furthermore, there is an evident disparity in intervention approaches, delivery techniques, and vaccination uptake measurement methods, though the less than 50% heterogeneity in vaccination uptake somewhat mitigates this concern. Most of the included studies focused on high-income countries, leaving a gap in understanding the effectiveness of digital interventions in low- and middle-income regions. This underscores an important gap in knowledge and points toward the necessity for further exploration in diverse geographic contexts. Additionally, the lack of studies assessing outcomes beyond a 2-year post-intervention period highlights the need for long-term evaluations.

Future studies of digital interventions to promote HVU might standardize outcome measures to compare with other interventions, and trial longitudinal designs. Large implementation trials that iteratively test models of user engagement in real-world settings are now needed to accelerate growth in this area. At scale, such trials can examine factors that mediate efficacy, to understand the conditions required to optimize the potency of these interventions.

Digital interventions with client reminder systems showed the largest overall improvements in HVU. Provider reminder interventions also exhibited substantial improvements in HVU. While other interventions like client education plus reminder and provider education showed significant improvements in HVU as well, the magnitude of their effects was comparatively smaller. The results suggest that client reminder and provider reminder interventions may be the most effective types of digital interventions for promoting HVU. We recommend that digital interventions with reminder system can, and should, be promoted and integrated into healthcare systems. However, there is also the need to extend and test their use in LMICs. This meta-analysis further indicates that client education intervention might be less effective than the other types of digital interventions for increasing HVU, raising important questions about the generalizability of vaccine education intervention in cancer prevention and the mechanisms underlying change.

## Methods

This systematic review protocol was registered in the PROSPERO database (CRD42023389004), and the review findings were conducted in accordance with the Preferred Reporting Items for Systematic Reviews and Meta Analyses (PRISMA) guidelines^72^ and the Cochrane Handbook for Systematic Reviews of Interventions^73^.

### Eligibility criteria

We included peer-reviewed articles published in English that met the PICOS strategy eligibility criteria:

Population (P): The study population consisted of children, adolescents, and young adults aged 9 to 26 who were eligible for the WHO-recommended HPV vaccine, as well as their parents or healthcare providers.

Intervention (I): The intervention intended to utilize digital technologies (e.g., SMS, Email, DVD, website, webinar, application) to remind or educate clients (children, adolescents, and young adults and/or parents) or healthcare providers about HPV vaccination. These interventions were designed and implemented with the specific intent of promoting HVU.

Comparison (C): Eligible studies were required to employ a control group, such as the usual condition or an alternative control. The meta-analysis excluded studies with only two digital interventions and no control group (i.e., non-inferiority trials).

Outcome (O): The studies examined the influence of digital interventions on HVU, including vaccination initiation and completion. We defined initiation as receiving a minimum of one dose of the HPV vaccine. We defined completion as receiving all of the recommended vaccine doses. Self-report (i.e., parent or patient) and provider-verified vaccination status were both acceptable methods for assessing vaccination coverage (i.e., medical records, immunization registries). Studies that provided only knowledge, attitudes, or intentions regarding HPV vaccination were excluded.

Study design (S): The studies were randomized controlled trials (RCTs).

### Search strategy

In January 2023, a systematic literature search was completed in PubMed, PsycInfo, Web of Science, and Cochrane Central Register of Controlled Trials. We used key words relating to (i) digital intervention (SMS, Email, DVD, website), (ii) HPV vaccine (e.g., HPV, cervical cancer vaccine) and (iii) vaccination uptake (e.g., initiation, completion, coverage). (search string for PubMed Supplementary Methods).

### Study selection

We examined the reference lists or relevant systematic reviews and primary studies that were identified. Titles and abstracts of retrieved reports were screened in Rayyan, independently by NC and TT to identify potentially eligible studies (90% overlap; disagreements resolved through discussion). The full-texts of these potentially eligible studies were independently assessed for meeting the criteria by research assistant, NC, and TT (85% overlap; disagreements resolved through discussion).

### Data extraction

For each study, we extracted information regarding (i) general study characteristics (e.g., year of publication), (ii) intervention characteristics (e.g., whether the intervention was client education, client reminder, provider education, or provider reminder), (iii) sample characteristics (e.g., children’s age), and (iv) data for calculation of effect sizes (i.e., sample size, vaccination uptake for both groups). In instances where studies reported both intention-to-treat and per-protocol analyses, we utilized the intention-to-treat data due to its inherent advantages in preserving the randomized treatment assignment and accounting for potential deviations from the protocol. By including all participants as originally assigned, regardless of adherence or protocol violations, intention-to-treat analysis provides a more conservative and unbiased estimate of treatment effects. Before entering data into statistical software, sample sizes for cluster sampling studies were reduced using the reported design effect and intracluster correlation coefficient^74^. Microsoft Excel was used to organise extracted data from included studies. All data items were coded by research assistant, NC, and TT with excellent reliability (84% to 100% agreement; mean per item 95%). If data was not retrievable from publication, study authors were contacted for clarification.

### Statistical analysis

We calculated the effect size OR, 95% confidence interval (95%CI), and P-value (P) for each comparison based on the post-assessment HVU (vaccination initiation or vaccine series completion) representing the difference between the two groups (digital intervention group versus control group) at post-assessment. Due to the anticipated heterogeneity between trials, a random effects model was used for all analyses. If trials were multi-armed and reported two comparisons to one, the sample size was divided to avoid inflating the power. Specifically, half of the total sample size was allocated to the control arm, while the remaining half was evenly distributed among the two comparison arms. This approach ensures unbiased estimation of treatment effects and is commonly employed in meta-analyses to account for the unbalanced nature of such trials^73^.

We log transformed the ORs, combined them using random-effects meta-analysis, and then exponentiated the pooled result to yield a pooled OR. Statistical heterogeneity was reported using the *I*^2^ statistic. We tested if findings were different if we only included trials providing client education, client reminder, the combination of client education plus reminder, provider education, provider reminder^75^ by using subgroup analysis. The subgroup analyses were conducted according to the mixed-effect model, in this model subgroups are pooled with the random-effects model while tests for significant differences between subgroups are conducted with the fixed-effects model. We included all relevant effect sizes and dealt with their dependency by conducting meta-analysis in Comprehensive Meta-Analysis Software (CMA) 3.0^76^. We conducted subgroup analyses using the CMA software, which allows for the exploration of differences in effect sizes across subgroups. This approach involves conducting separate meta-analyses within each subgroup and comparing the results to assess potential subgroup effects. The CMA software allowed us to conduct separate meta-analyses for each selected effect size, while appropriately handling the dependency between effect sizes from the same study. CMA software includes a robust method to address the presence of multiple effect sizes per study and deals with the dependency among these effect sizes during the meta-analysis process. We ensured that all relevant effect sizes were included and appropriately accounted for their dependencies.

### Quality assessment

Two authors (TT and NC) independently used Cochrane’s Risk of Bias (ROB) 2.0 tool^77^ for assessing risk of bias to assess included RCTs and their respective protocols and trial registry records for risk of bias in five domains: (1) bias arising from the randomization process, (2) bias due to deviations from intended interventions, (3) bias due to missing outcome data, (4) bias in measurement of the outcome and (5) bias in selection of the reported result. The ROB 2.0 tool employs a comprehensive and standardized algorithm that considers specific criteria within each domain to calculate the overall risk of bias. Disagreements between the authors were resolved through discussion. We assessed publication bias by visually examining a funnel plot for the main outcome and conducting Egger’s test^78,79^. To obtain an estimation of the pooled effect when accounting for missing studies, the Duval and Tweedie trim-and-fill analysis was performed in ref. ^79^.

### Reporting summary

Further information on research design is available in the Nature Research Reporting Summary linked to this article.

### Supplementary information

Supplementary Material

Reporting Summary

## Data Availability

The data used in this meta-analysis are available upon request. Researchers interested in accessing the data can contact us via email. We are committed to promoting transparency and facilitating further research in the field of HPV vaccination uptake. Therefore, we welcome inquiries regarding the data used in this study and will provide the requested information to the best of our ability.
