# Supplementary material for: Systematic review and meta-analysis comparing educational and reminder digital interventions for promoting HPV vaccination uptake
Source: NPJ Digit Med. 2023 Aug 29;6:162. doi: 10.1038/s41746-023-00912-w (PMC10465590; doi:10.1038/s41746-023-00912-w)
Supplement: Supplementary file 1 — Supplementary Material [file 41746_2023_912_MOESM1_ESM.pdf]

Supplement:

Supplementary Figure 1 Risk of bias plot over all studies

Supplementary Figure 2 Risk of bias plot per study

Supplementary Figure 3 Forest plot of pooled ORs over all studies

Supplementary Table 1 Study characteristics

Supplementary Methods: Search String Pubmed

Supplementary Figure 1 Risk of bias plot over all studies

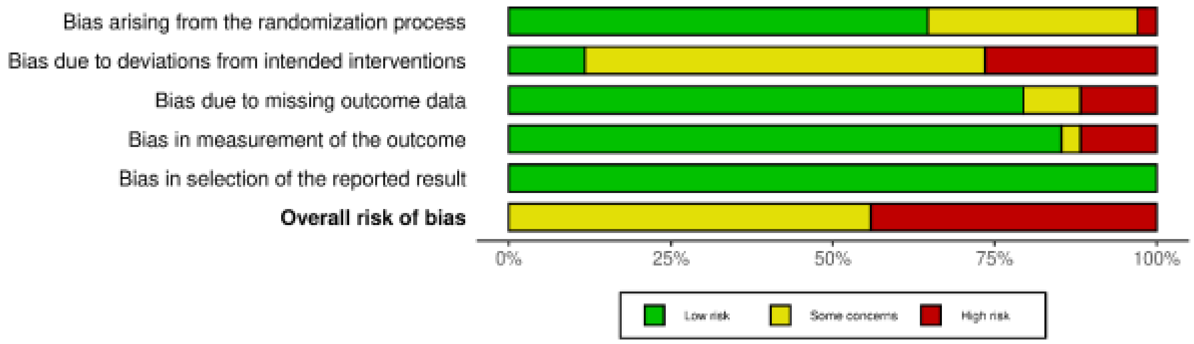

Supplementary Figure 2 Risk of bias plot per study

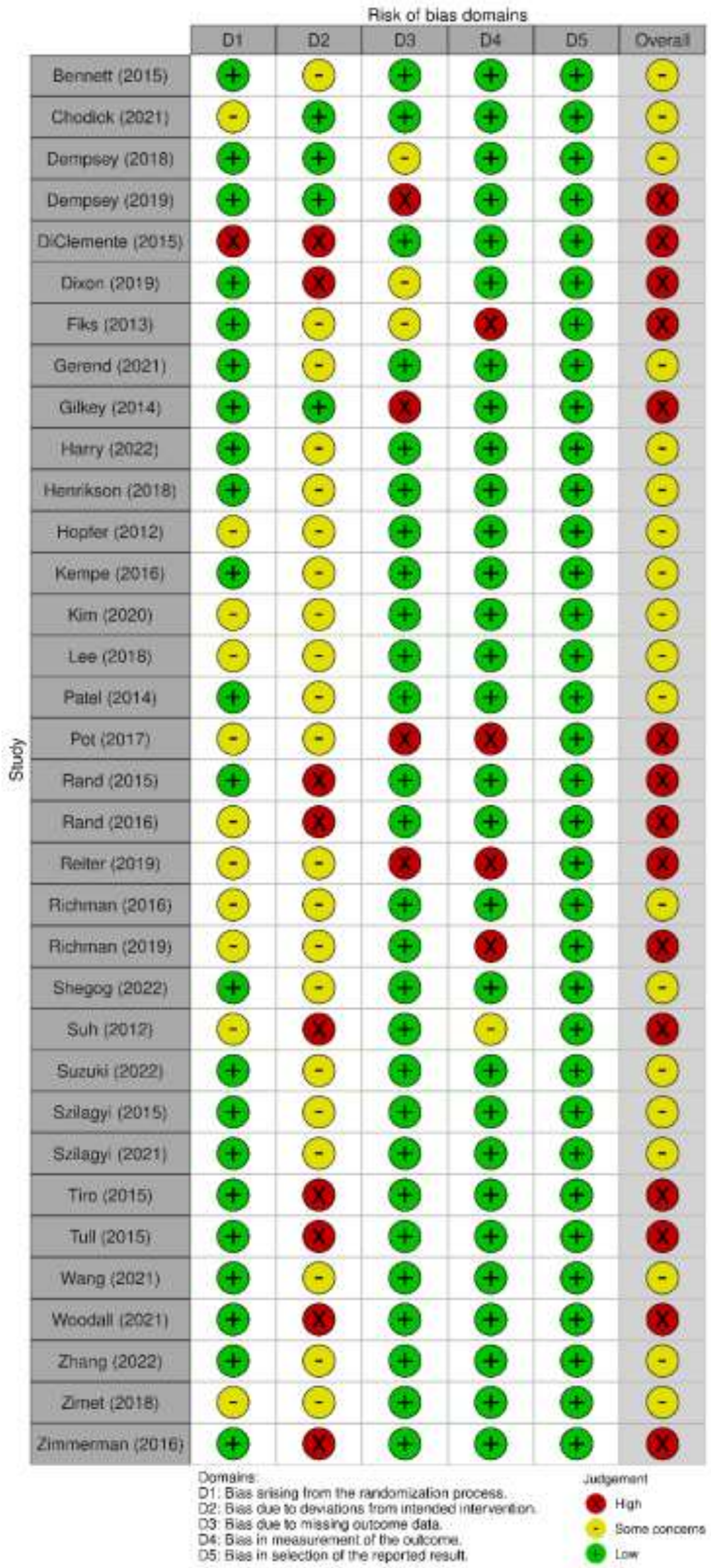

Supplementary Figure 3 Forest plot of pooled ORs over all studies

Group by COMPARE	Study name	Odds ratio	Lower limit	Upper limit	Z-Value	p-Value
CE	Bennet,2015	0.888	0.511	1.543	-0.422	0.673
CE	Chodick,2021	1.012	0.946	1.082	0.343	0.731
CE	Dempsey,2019,1	1.024	0.244	4.302	0.033	0.974
CE	Dempsey,2019,2	1.037	0.247	4.356	0.050	0.960
CE	DiClemente,2015	1.045	0.446	2.451	0.102	0.919
CE	Hopfer,2011	2.073	1.048	4.101	2.095	0.036
CE	Kim,2020	2.395	0.576	9.951	1.202	0.230
CE	Lee,2018	1.000	0.108	9.229	0.000	1.000
CE	Reiter,2018	2.381	1.173	4.833	2.402	0.016
CE	Suzuki,2022	0.903	0.646	1.263	-0.597	0.551
CE	Zhang,2022	1.114	0.420	2.952	0.217	0.828
CE	Pooled	1.084	0.916	1.282	0.941	0.347
CR	Fiks,2013,3	1.618	1.143	2.291	2.715	0.007
CR	Henrikson,2018	1.339	1.009	1.779	2.019	0.043
CR	Kempe,2016	1.936	1.062	3.531	2.157	0.031
CR	Patel,2014	1.073	0.654	1.760	0.279	0.781
CR	Rand,2015	1.273	1.062	1.526	2.614	0.009
CR	Rand,2016	2.157	1.428	3.259	3.651	0.000
CR	Suh,2012	1.760	1.257	2.463	3.296	0.001
CR	Tiro, 2015	1.058	0.800	1.398	0.393	0.694
CR	Tull,2019,2	1.358	1.028	1.794	2.154	0.031
CR	Pooled	1.411	1.225	1.626	4.772	0.000
CRCE	Gerend,2021	3.428	1.166	10.077	2.239	0.025
CRCE	Pot,2017	1.026	0.930	1.132	0.517	0.605
CRCE	Richman,2016	1.033	0.678	1.573	0.150	0.881
CRCE	Richman,2019	1.000	0.606	1.650	0.000	1.000
CRCE	Shegog,2022	1.383	0.818	2.336	1.211	0.226
CRCE	Tull,2019,1	1.368	1.036	1.806	2.207	0.027
CRCE	Wang,2021,1	2.512	1.122	5.622	2.240	0.025
CRCE	Wang,2021,2	1.565	0.678	3.616	1.049	0.294
CRCE	Woodall,2021	2.143	0.820	5.603	1.554	0.120
CRCE	Pooled	1.288	1.043	1.589	2.356	0.018
PE	Dempsey,2018	1.188	0.850	1.659	1.009	0.313
PE	Gilkey,2014	1.528	0.880	2.652	1.507	0.132
PE	Szilagyi,2021	1.131	0.975	1.310	1.630	0.103
PE	Zimmerman,2017	1.342	0.970	1.856	1.778	0.075
PE	Pooled	1.183	1.048	1.336	2.708	0.007
PR	Dixon,2019	1.845	1.236	2.755	2.996	0.003
PR	Fiks,2013,1	1.898	1.334	2.700	3.560	0.000
PR	Fiks,2013,2	1.206	0.856	1.700	1.072	0.284
PR	Harry,2022,1	1.205	0.815	1.781	0.935	0.350
PR	Harry,2022,2	0.814	0.535	1.238	-0.962	0.336
PR	Szilagui,2015	1.000	0.624	1.601	-0.001	0.999
PR	Zimet,2018,1	1.975	1.239	3.150	2.860	0.004
PR	Zimet,2018,2	1.761	1.030	3.010	2.068	0.039
PR	Pooled	1.391	1.106	1.751	2.817	0.005
Overall	Pooled	1.250	1.163	1.343	6.097	0.000

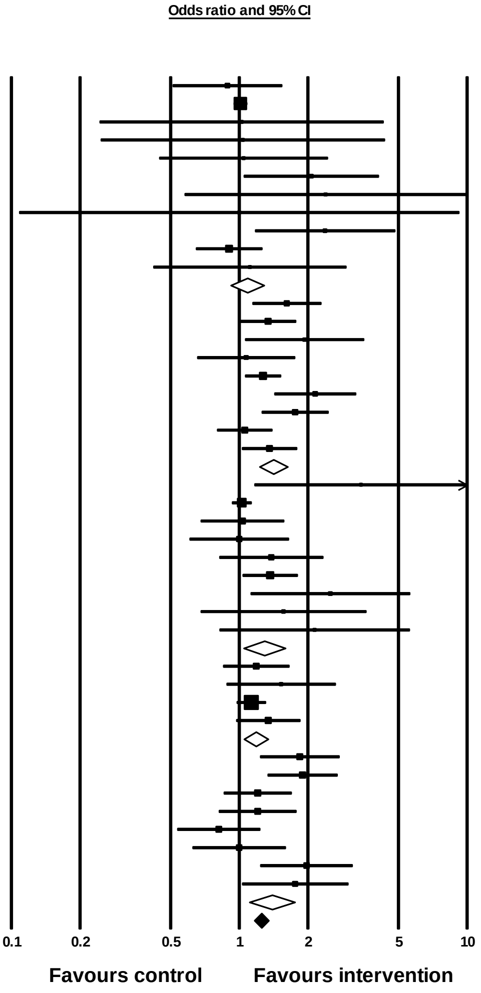

Overall ORs across all interventions, client education (CE), client reminder (CR), client education plus reminder (CRCE), provider education (PE), provider reminder (PR) interventions

Supplementary Table 1 Study characteristics

Study	Country	N	Population	Child age	Intervention	Platform	Type	Comp	Duration	Outcomes
Bennett 2015[44]	US	661	Female university students	21.0 (18-26)	MeFirst (a tailored, online educational intervention)	Website	CE	AT	3 m	initiation
Chodick 2021[45]	Israel	21,592	Mothers	14.0	Facebook campaign	Facebook	CE	UC	2 m	initiation
Dempsey 2018[46]	US	43,132	Adolescents	12.6 (11-17)	Health Care Profe ssional Communication Training Inter vention	Webinar	PE	UC	1 y	initiation
Dempsey 2019[47]	US	1,294	Latino adolescents	(9-26)	CHICOs (iPad-based, tailored messaging intervention)	Website +iPad	CE	UC	1 y	initiation
DiClemente 2015[48]	US	216	African American female	16.5 (14-18)	Girl OnGuard (computer-delivered educational intervention)	DVD	CE	AT	7 m	initiation
Dixon 2019[42]	US	1,596	Providers	none 11-12	The child health improvement through computer automation (CHICA) clinical decision support system	EHR, CDS	PR	UC	2 w	initiation
Fiks 2013[49]	US	22,486	Providers	11-17	Clinician focus intervention	EHR, CDS	PR	UC	1 y	initiation
Gerend 2021[50]	US	150	Men who have sex with men	18-25	Text-messaging based HPV vaccination intervention	SMS	CRCE	AT	9 m	initiation
Gilkey 2014[51]	US	107,443	Providers	11-18	Webinar-delivered AFIX	Webinar	PE	UC	1 y	initiation
Harry 2022[52]	US	6,274	Providers	18-26	Clinical decision support with shared decision making tools	EHR, CDS	PR	UC	1 y	initiation
Henrikson et 2018[53]	US	1,805	Parents of adolescents	11.0 (10-12)	Outreach and automated reminders	SMS	CR	UC	120 d	initiation
Hopfe 2012[35]	US	404	College women	21.0 (18-26)	Narrative intervention	DVD	CE	AT	2 m	initiation
Kempe 2016[36]	US	1,422	Adolescents	13.0 (11-17)	Preference-based recall	SMS, e-mail, auto-dialer	CR	UC	1 y	completion
Kim 2020[54]	US	104	Korean American college women	21.7 (18-26)	A storytelling intervention in a mobile, web-based platform	Website	CE	AT	2 m	initiation
Lee 2018[55]	US	38	Khmer mothers and daughters	15.3 (14-17)	Storytelling narrative intervention videos	Videos	CE	AT	1 m	initiation
Patel 2014[56]	US	365	Women	23.2 (19-26)	Preference-based recall	SMS, e-mail, phone, Facebook message	CR	UC	32 w	completion
Pot 2017[39]	The Netherlands	8,062	Mothers of invited girls	12.5 (12-13)	a tailored, online educational intervention with virtual assistants	Website	CRCE	UC	2 w	initiation
Rand 2015[38]	US	3,812	Publicly insured adolescents	11-16	A managed care organization-generated text message	SMS	CR	AT	1 y	initiation
Rand 2016[37]	US	977	Parents	13.7 (11-17)	Text message reminders	SMS	CR	UC	1 y	completion

Reiter et 2019[57]	US	150	Young gay and bisexual men	(18-25)	Outsmart HPV intervention	SMS, email	CE	AT	3 m	initiation
Richman 2016[40]	US	264	College students	20.7 (18-26)	Text and e-message reminders	SMS, e-mail	CRCE	UC	7 m	completion
Richman 2019[41]	US	257	Uninsured or Medicaid-insured parents	12.0 (9-17)	Text and email reminders	SMS, email	CRCE	UC	7 m	initiation
Shegog 2022[27]	US	375	Parents	(13.5) 11-17	HPVCancerFree App	App	CRCE	UC	5m	initiation
Suh 2012[58]	US	1,600	Adolescents	14.1 (11-18)	Reminder/Recall	Autodialer phone call	CR	UC	12 m	initiation
Suzuki 2022[34]	Japan	2,175	Parents	11-18	Web-based cervical cancer survivor's story	Website	CE	UC	3 m	initiation
Szilagyi 2015[59]	US	7,040	Providers	11-18	Provider prompts	EHR	PR	UC	none	initiation
Szilagyi 2021[60]	US	29,261	Providers	11-17	Online communication training	Website	PE	UC	6 m	initiation
Tiro 2015[61]	US	814	Providers	11-18	Safety-Net clinic	EHR	PR	AT	12 m	initiation
Tull 2015[62]	Australia	4,386	Parents of secondary school students	13.2 (11-18)	SMS reminders	SMS	CRCE	UC	1 y	initiation
Wang 2021[63]	China	624	Men who have sex with men	18-45	Web-based intervention	Website	CRCE	AT	24 m	completion
Woodall 2021[64]	US	82	Parents of female adolescents	11-14	Vacteen.org (a mobile web app)	Web app	CRCE	UC	3 m	initiation
Zhang 2022[65]	China	946	College girl student	18.99	Web-based health education	Website	CE	AT	1 m	initiation
Zimet 2018[43]	US	648	Providers	11-13	The child health improvement through computer automation (CHICA) clinical decision support system	EHR, CDS	PR	UC	1 y	initiation
Zimmerman 2016[32]	US	10861	Providers	11-17	The 4 pillars practice transformation program	Website	PE	UC	9 m	initiation

Platform; EHR, electronic health record; CDS, clinical decision system; Comp, comparison condition; AT, alternative treatment; UC, usual care; Type: CE, client education; CR, client reminder; CRCE, client education plus client reminder; PE, provider education; PR, provider reminder; Duration: d, days; w, weeks; m, months

Supplementary Methods Search string PubMed

Search	Query	Items found
#4	#1 AND #2 AND #3	631
#3	"Cell Phone]"Mesh [OR EMR]tiab [OR EHR]tiab [OR Computer]*Mesh [OR "Mobile Applications]"Mesh [OR "Internet]"Mesh [OR mhealth]*tiab [OR m health]*tiab [OR telehealth]*tiab [OR tele health]tiab [OR ehealth]*tiab [OR e-health]*tiab [OR app]tiab [OR apps]tiab [OR smartphone]*tiab [OR smart phone]*tiab [OR phone application]*tiab [OR telephone application]*tiab [OR health application]*tiab [OR ipad]tiab [OR ipads]tiab [OR sms]tiab [OR mms]tiab [OR text messag]*tiab [OR texting]tiab [OR ussd]tiab [OR mhapps]tiab [OR iphone]*tiab [OR android]tiab [OR whatsapp]*tiab [OR social media]*tiab [OR social network]*tiab [OR facebook]*tiab [OR instagram]*tiab [OR webinar]tiab [OR twitter]*tiab [OR youtube]tiab [OR digital]*tiab [OR mobile]tiab [OR cell email]tiab [	517,691
#2	"Vaccination]"Mesh [OR vaccin]*tiab [OR immunizati]*tiab [OR immunisati]*tiab [	540,720
#1	human papilloma vir]*tiab [OR hpv]tiab [OR hpvs]tiab [OR papillomavir]*tiab [	64,266
